# Circadian rhythm promotes the biomass and amylose hyperaccumulation by mixotrophic cultivation of marine microalga *Platymonas helgolandica*

**DOI:** 10.1186/s13068-022-02174-2

**Published:** 2022-07-06

**Authors:** Qianwen Shi, Cheng Chen, Tingwei He, Jianhua Fan

**Affiliations:** 1 State Key Laboratory of Bioreactor Engineering, East China University of Science and Technology, 130 Meilong Road, Shanghai, 200237 People’s Republic of China; 2 Department of Applied Biology, East China University of Science and Technology, Shanghai, 200237 People’s Republic of China; 3 Department of Bioengineering, East China University of Science and Technology, Shanghai, 200237 People’s Republic of China; 4 School of Chemistry and Chemical Engineering, Shihezi University, Shihezi, 832003 People’s Republic of China

**Keywords:** Microalgae, Starch, Circadian rhythm, Mixotrophy, Amylose

## Abstract

**Background:**

Microalgal starch can be exploited for bioenergy, food, and bioplastics. Production of starch by green algae has been concerned for many years. Currently commonly used methods such as nutrient stress will affect cell growth, thereby inhibiting the production efficiency and quality of starch production. Simpler and more efficient control strategies need to be developed.

**Result:**

We proposed a novel regulation method to promote the growth and starch accumulation by a newly isolated Chlorophyta *Platymonas helgolandica*. By adding exogenous glucose and controlling the appropriate circadian light and dark time, the highest dry weight accumulation 6.53 g L^−1^ (Light:Dark = 12:12) can be achieved, and the highest starch concentration could reach 3.88 g L^−1^ (Light:Dark = 6:18). The highest production rate was 0.40 g L^−1^ d^−1^ after 9 days of production. And this method helps to improve the ability to produce amylose, with the highest accumulation of 39.79% DW amylose. We also discussed the possible mechanism of this phenomenon through revealing changes in the mRNA levels of key genes.

**Conclusion:**

This study provides a new idea to regulate the production of amylose by green algae. For the first time, it is proposed to combine organic carbon source addition and circadian rhythm regulation to increase the starch production from marine green alga. A new starch-producing microalga has been isolated that can efficiently utilize organic matter and grow with or without photosynthesis.

**Supplementary Information:**

The online version contains supplementary material available at 10.1186/s13068-022-02174-2.

## Background

Microalgae refers to a class of low-grade, autotrophic aquatic microorganisms, a relatively low-level group of microscopic single-cell populations. Microalgae can use light and carbon dioxide for photosynthesis, release oxygen and synthesize organic substances at the same time. It is one of the biological groups with the highest photosynthetic efficiency in nature. Microalgae can provide many metabolites with commercial value, such as lipid, starch, polysaccharide and phycobiliprotein [1, 2]. Starch is the main carbohydrate storage method of many microalgae, and it is not only important caloric component of food, but also industrial material [3]. In Chlorophyta, starch accumulation is especially abundant. Since the structure of starch produced by microalgae is similar to plant starch, it is often considered as a substitute for plant starch in food, bio-based chemicals (biological plastics, etc.), and bioenergy (bioethanol, biohydrogen, etc.) [4–6]. Due to the rapid growth of microalgae, high photosynthetic efficiency and CO_2_ fixation capacity, as well as the saving of arable land, it has attracted more attention in recent years [6, 7].

The accumulation of starch in microalgae can be promoted by various means. In the past few years, the commonly used methods are mainly the regulation of nutrient elements, such as changes in nitrogen, phosphorus, and sulfur [8–10]. Nitrogen deficiency/limitation was the most commonly used strategy in Chlorophyta, and could often make the starch accumulation reach more than 50% of the dry weight (DW) [11]. However, nutritional stress can inhibit the growth of algae cells and limit the rate of starch production. In recent years, it was found that microalgae cells cultured under mixotrophic or heterotrophic conditions with the addition of exogenous carbon sources can accumulate large amount of useful metabolites, including starch [12]. It has been found that many kinds of Chlorophyta can grow mixotrophically/heterotrophically [13, 14]. The available carbon sources include glucose, acetic acid/acetate, glycerol and so on. The addition of high-concentration acetate in *Chlamydomonas reinhardtii* culture results in increased dry weight and accumulation of multiple biomass components (including starch, fatty acids, proteins, etc.) compared with adding low-concentration acetate [15]. Another most often used is glucose. However, the use of glucose for long-term culture will increase the cost, not to mention the risk of infection [14, 16, 17].

The production rate is very important in starch production. It can be achieved by increasing the growth rate of algae. Optimizing the culture medium and culture conditions is the most used method [10, 18, 19]. Usually in laboratory culture, in order to pursue continuous growth of cells, continuous light is often adopted, while ignoring the influence of natural laws such as cell cycle and circadian rhythm. Due to the energy storage function of starch in the cell cycle of microalgae, the simultaneous cultivation of some Chlorophyta can promote the accumulation of starch [20–22]. At the same time, the combined effect of light conditions and external carbon sources cannot be ignored. Study in *Chlorella zofingiensis* showed that light and non-light conditions can regulate cells to converse added glucose to starch or lipids [23]. Up to now, research on cell cycle and starch production mainly focuses on its biological value, and few studies have used light–dark cycles as a control method to promote starch accumulation [11].

*Platymonas helgolandica* var. tsingtaoensis (*P. helgolandica*) is a unique species of Chlorophyta that grows in the coastal waters of China (Additional file 1: Fig. S1). It is currently commonly used as a bait for shrimp and shellfish. This study aimed to develop a new and simple regulation method to produce high-quality starch using *P. helgolandica*, and to provide new ideas for optimizing the starch production efficiency of green algae. This study firstly confirmed that *P. helgolandica* can use glucose for both mixotrophic and heterotrophic culture, and for the first time through the addition of both exogenous carbon sources and the regulation of circadian rhythms, the growth and starch (mostly amylose) production capacity of *P. helgolandica* was greatly improved. The produced starch can be used as a potential substitute for food starch, and can also be used to produce clean energy such as bioethanol and biohydrogen.

## Results and discussion

### Glucose benefits growth and starch accumulation in *P. helgolandica*

**Fig. 1:**
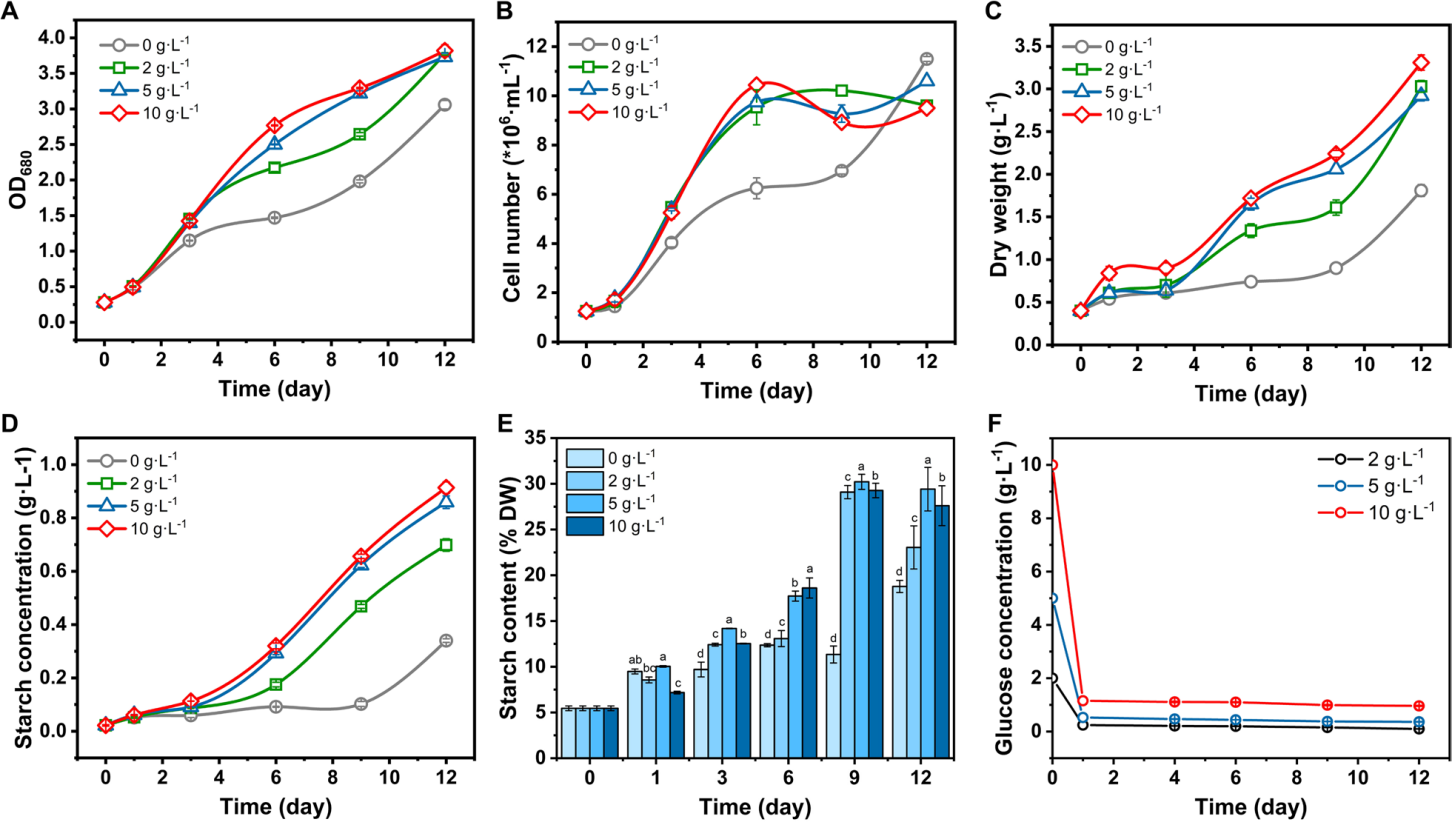
The effect of adding glucose on the growth performance of *P. helgolandica*. **A** Absorbance value of 680 nm; **B** cell number; **C** dry weight; **D** starch concentration in cultures; **E** the ratio of starch accumulation in cells to dry weight; **F** glucose consumption in culture supernatant

In order to explore whether glucose can promote the growth of *P. helgolandica*, glucose was added to the culture to a final concentration of 0, 2, 5, and 10 g L^−1^, and the growth parameters were measured under mixotrophic culture (continuously illuminated) during the cultivation process (Fig. 1).

In the early growth period, the higher the glucose concentration was, the more obvious the promotion of the growth rate was (Fig. 1A–C). In the late growth period, the approximate absorbance and dry weight can be achieved. The dry weight accumulation was highest in the presence of 10 g L^−1^ glucose, which can reach 3.31 g L^−1^ (Fig. 1C). According to the cell number curve, in the early stage of culture, the accumulation of dry weight mainly come from the increase of cell number; however, in the later stage of culture (after 6 days), the increase of cell number slowed down, whereas the dry weight continued to increase. The change of glucose concentration in the culture supernatant was measured (Fig. 1F). After one day of culture, the glucose concentration changed drastically (reduced by 90%), and the change was relatively small in the later period of the culture. These results prove that glucose can indeed promote the growth of *P. helgolandica*.

In addition to promoting growth, the presence of glucose also promotes the accumulation of carbohydrates (mainly starch). We determined the changes in starch accumulation during the growth of *P. helgolandica* (Fig. 1D, E). During the culture process, the concentration of starch in the culture continued to rise. The starch concentration in the control group (glucose free) increased very slowly during the rapid growth period (1–6 days). The three groups with glucose accumulated starch while growing rapidly, indicating that glucose provides extra energy. The cell number continued to increase rapidly within 6 days, and slowed down after 6 days, but the dry weight continued to increase. The proportion of starch in dry weight gradually increases with growth time, and can accumulate up to 30.21% of dry weight (5 g L^−1^ glucose), increasing by 60% (*p* < 0.05) compared to the control group. The concentration of starch of the 10 g L^−1^ glucose group is the highest (0.91 g L^−1^), increasing by 167% compared to the control group. After 12 days, the dry weight stopped increasing and starch accumulation reached its peak.

*Tetraselmis *spp. is considered as a dominant group of green microalgae to produce starch. Its allied species *T. subcordiformis* tends to accumulate starch when exposed to stress, compound stimulation, etc. [8]. According to previous reports, other species of the genus *Tetraselmis* can also use glucose for growth. In general, the increase in glucose concentration can better promote the dry weight accumulation of *Tetraselmis*. However, the promotion effect becomes insignificant after a certain concentration (≥ 10 g L^−1^) [14]. In other species, the addition of glucose will increase the accumulation of different biomass, such as lipids [24] and proteins [25]. However, *P. helgolandica* accumulated starch relatively uniquely.

**Fig. 2:**
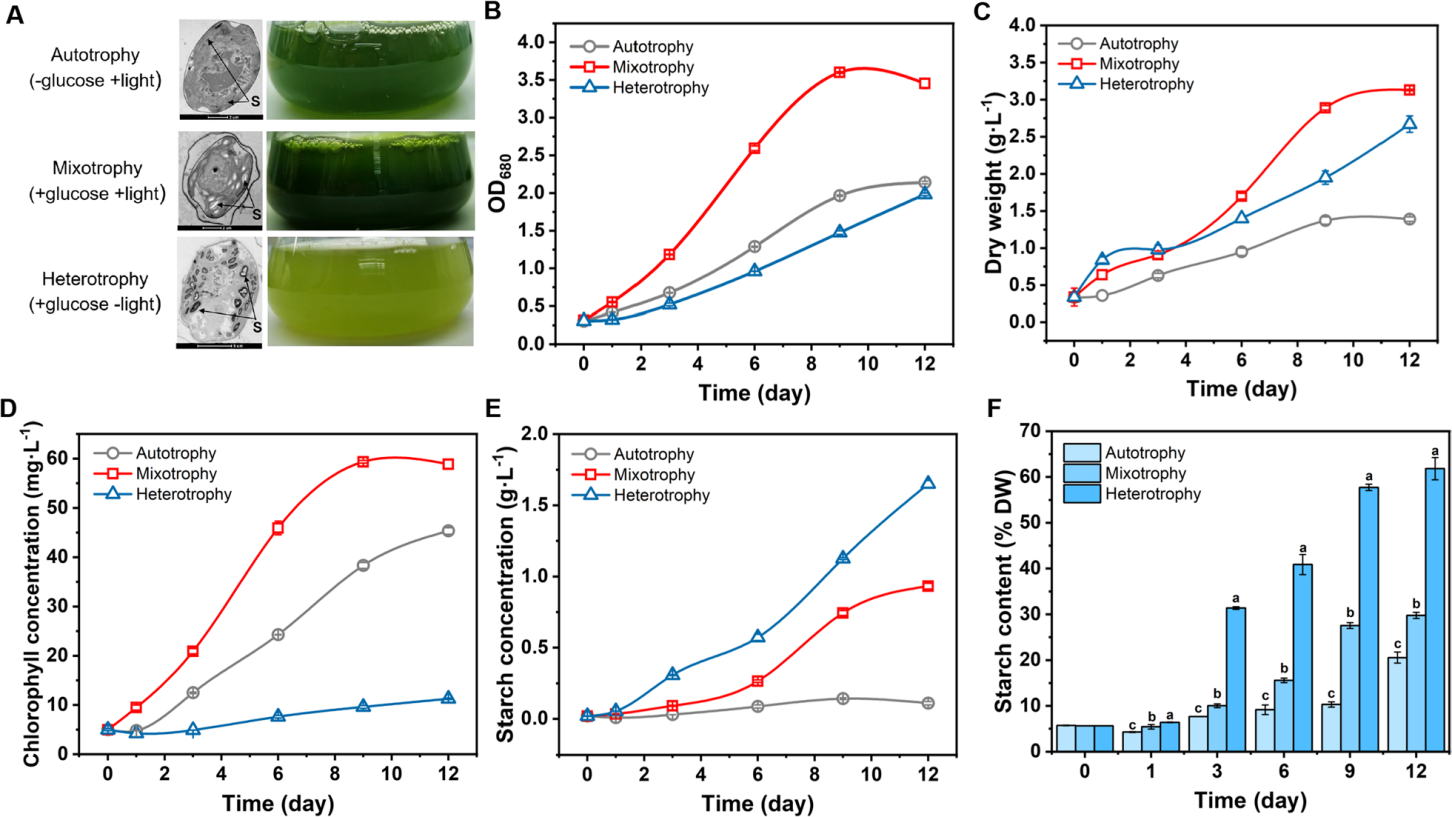
The effect of different culture modes on the growth of *P. helgolandica*. **A** Transmission electron microscopy (TEM) pictures of cells under different culture conditions and colors of cultures, scare bar for autotrophy and mixotrophy is 2 μm, for heterotrophy is 5 μm; **B** absorbance value of 680 nm; **C** dry weight; **D** chlorophyll concentration; **E** starch concentration in cultures; **F** the ratio of starch accumulation in cells to dry weight, lower-case letters represent significant differences (*p* < 0.05)

Glucose can be used as an exogenous organic substrate for the culture of *P. helgolandica*, and can strongly promote the growth and biomass accumulation. In the subsequent cultivation, 10 g L^−1^ glucose was added to the KWF medium. Glucose can support the growth of *P. helgolandica* in different culture modes (Fig. 2). Adding glucose for mixotrophic cultivation can significantly increase the growth rate of *P. helgolandica* and accumulate a higher dry weight (3.13 g L^−1^). The heterotrophic cultivation with continuous darkness can also promote the dry weight accumulation (2.67 g L^−1^). Compared with the continuous light autotrophic cultivation, the dry weight increased by 125% and 92%, respectively. Under heterotrophic conditions, the number of algae cells is relatively small, but the cell size and weight increased, resulting in an increase in dry weight accumulation (Fig. 2A–C). This should be attributed to the fact that the algae cells accumulated more starch (Fig. 2A). It can be seen that the size and number of starch granules in the cells under mixotrophic culture is between that of the autotrophic and heterotrophic cultured cells. In addition, the cell morphological changes during mixotrophic and heterotrophic culture, and we observed that the cell wall became not as smooth as autotrophic cultured cells, and even a multi-layered wall structure appeared (Fig. 2A). This may reflect the transformation of the cells into a special nutritive cell morphology called akinete [26].

The final starch accumulation can reach 61.82% of the dry weight, which is 102% higher than continuous light mixotrophic cultivation and 201% than autotrophic cultivation (Fig. 2F). The concentration of starch in the culture can reach 1.65 g L^−1^ (Fig. 2E). The algae culture under heterotrophic conditions was yellow-green (Fig. 2A), and the determination of chlorophyll reflects that the algae cells under heterotrophic cultivation contain very little chlorophyll (Fig. 2D).

### The regulation of circadian rhythm affects the growth and promotes starch hyperaccumulation in *P. helgolandica*

**Fig. 3:**
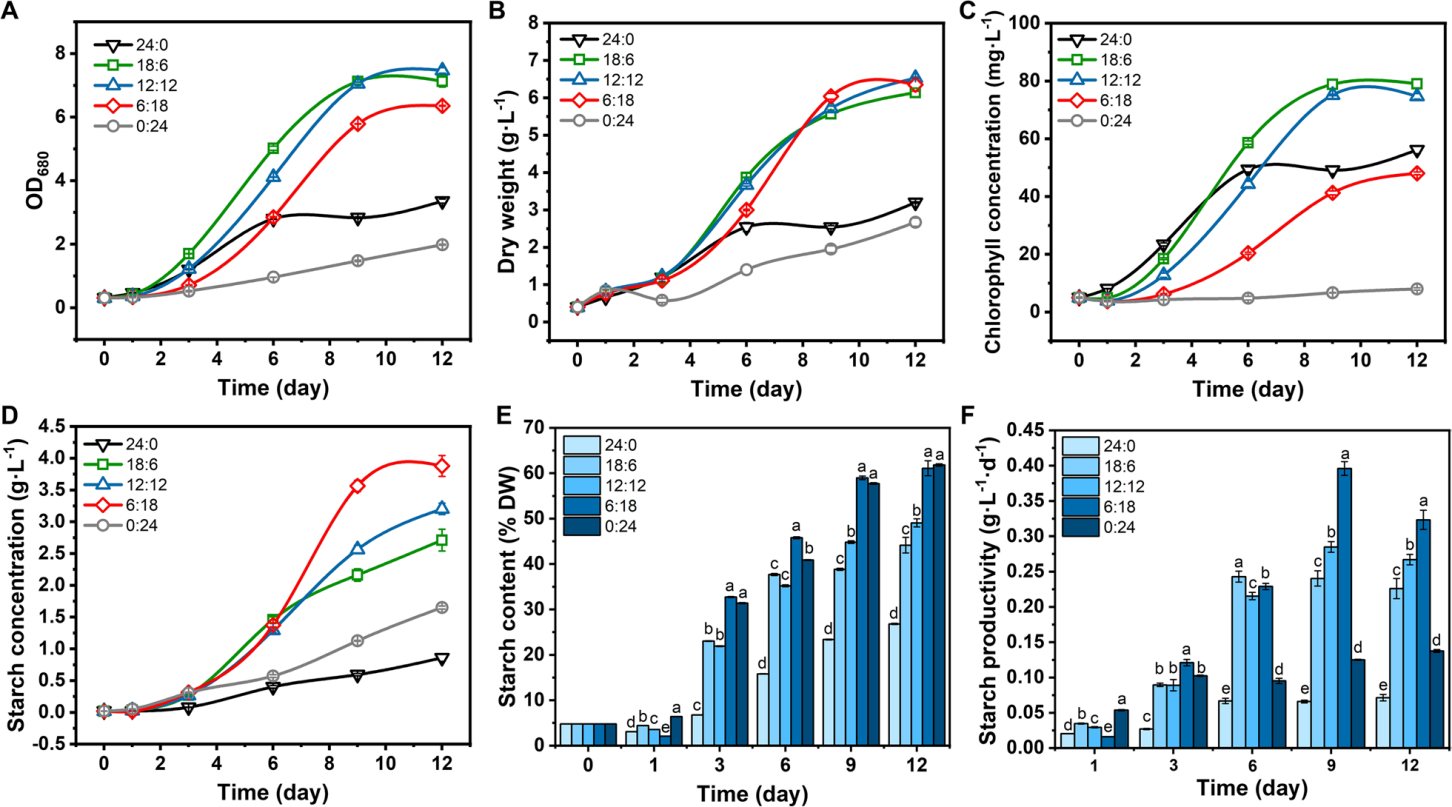
The effect of circadian rhythm on the growth and starch accumulation of *P. helgolandica*. **A** Absorbance value of 680 nm; **B** dry weight; **C** chlorophyll concentration; **D** starch concentration in cultures; **E** the ratio of starch accumulation in cells to dry weight; **F** starch productivity on different days; lower-case letters represent significant differences (*p* < 0.05)

Glucose can be used as an exogenous organic carbon source to promote the growth and starch accumulation of *P. helgolandica* under different culture conditions. Although in the heterotrophic cultivation, the algae cells can accumulate starch by about 60% of the dry weight, which is relatively high, the cells are not growing fast enough. Therefore, the rate of starch production is not high enough, which greatly limits the use of this algae to produce starch. The circadian rhythm is an important factor affecting the growth of green algae. Therefore, different circadian rhythms (*L*:*D* = 18:6, 12:12, 6:18) were used for the cultivation of *P. helgolandica*, and samples were taken over time to determine growth and related parameters (Fig. 3). All experimental groups in this section are cultured with 10 g L^−1^ glucose.

The existence of circadian rhythm greatly promoted the growth of *P. helgolandica* (Fig. 3A, B). Among them, *L*:*D* = 12:12 (h) has the strongest promotion of cell population. The three rhythm patterns can promote the accumulation of dry weight, the highest of which is the 12:12 group. Under this rhythm the dry weight can reach 6.53 g L^−1^, which is 104% higher than the dry weight accumulated under continuous illumination. When the circadian rhythm is *L*:*D* = 6:18 (h), the algae can accumulate the most starch. It accounts for 61.09% of dry weight, which was 128% higher than 24:0 group (Fig. 3E). The highest starch concentration and highest yield are relatively 3.88 g L^−1^ (day 12) and 0.40 g L^−1^ d^−1^ (day 9), 351% and 460% improved separately (Fig. 3D–F). It proves that the circadian rhythm is an important factor to promote the growth and the accumulation of starch in *P. helgolandica*. The kinetic parameters for growth and starch accumulation are listed in Table 1.

**Table 1:** The kinetic parameters for growth and starch accumulation of *P. helgolandica* cultures under different cultivation modes (mean ± SD, *n* = 3)

	Autotrophy	Mixotrophy	Mixotrophy under circadian cycle			Heterotrophy
	24:0 (-Glc)	24:0	18:6	12:12	6:18	0:24
X1_max_ (g_TS_ L^−1^)	0.34 ± 0.01	0.91 ± 0.02	2.71 ± 0.17	3.20 ± 0.09	3.88 ± 0.16	1.65 ± 0.02
X2_max_ (g_Am_ L^−1^)	0.17 ± 0.00	0.44 ± 0.01	1.64 ± 0.08	2.09 ± 0.06	2.53 ± 0.11	0.83 ± 0.01
X3_max_ (g_DW_ L^−1^)	1.81 ± 0.07	3.13 ± 0.01	6.14 ± 0.12	6.53 ± 0.03	6.35 ± 0.17	2.67 ± 0.11
Y (g_Am_ g_Glc_^−1^)	/	0.05 ± 0.01	0.18 ± 0.00	0.23 ± 0.01	0.28 ± 0.01	0.09 ± 0.00
μ (D^−1^)	0.07 ± 0.01	0.85 ± 0.01	1.30 ± 0.04	1.31 ± 0.01	1.43 ± 0.02	0.76 ± 0.03
Q_X1max_ (g_TS_·L^−1^ D^−1^)	0.03 ± 0.00	0.08 ± 0.00	0.24 ± 0.02	0.28 ± 0.05	0.40 ± 0.08	0.14 ± 0.02
Q_X2max_ (g_Am_·L^−1^ D^−1^)	0.01 ± 0.00	0.04 ± 0.00	0.17 ± 0.02	0.19 ± 0.05	0.25 ± 0.06	0.07 ± 0.01

X1—total starch, X2—amylose, X3—dry weight, Y—yield of amylose on glucose, μ—growth rate, Q_X1max_—production rate of total starch, Q_X2max_—production rate of amylose

In order to further evaluate the quality of the starch produced, the content of amylose (Am) and amylopectin (Ap) in the starch was determined. Starch is the main storage carbohydrate in plants and some algae. Usually amylose accounts for < 35% of most natural starches, and the rest is amylopectin [27]. Amylose-rich starch is beneficial to lower digestive tract health when used as food [28], and is also suitable as a biodegradable plastic substitute [29]. We found that this strain of *P. helgolandica* naturally accumulates much amylose. Under continuous light autotrophic culture, the ratio of Am and Ap (Am/Ap) is usually close to 1:1 (amylose ratio ≈ 50% TS). After the addition of glucose, there was no significant difference in Am/Ap (*p* > 0.05) regardless of whether it is mixotrophic (continuous light) or heterotrophic (continuous darkness) cultured. When cultured under circadian rhythm with glucose added, Am/Ap increased significantly (*p* < 0.05) (Table 2). In *P. helgolandica*, a cycle of *L*:*D* = 6:18 (h) can carry out 39.79% DW of amylose. The yield of amylose on glucose could reach 0.28 g_Am_·g_Glc_^−1^ under this cycle (Table 1). It has long been reported in rice and other plants that the expression of *gbss*, a gene related to amylose synthesis, is related to carbon sources and circadian rhythms. Sugar, especially glucose, strongly promotes the expression of *gbss.* At the same time, an appropriate photoperiod will also promote the expression of *gbss* [30].

**Table 2:** Am/Ap ratio, and Am or Ap content (%DW) of *P. helgolandica* cultures under different cultivation modes (mean ± SD, *n* = 3)

Rhythm (L:D)	Am/Ap						C_Am/DW_ (%DW)						C_Ap/DW_ (%DW)				
	Day1	Day3	Day6	Day9	Day12		Day1	Day3	Day6	Day9	Day12		Day1	Day3	Day6	Day9	Day12
24:0 (-Glc)	1.60 ± 0.06^d^	1.12 ± 0.04^c^	0.85 ± 0.13^d^	1.02 ± 0.23^b^	1.03 ± 0.04^c^		5.82 ± 0.22^a^	5.13 ± 0.79^c^	5.64 ± 0.45^e^	5.60 ± 0.33^e^	9.53 ± 0.05^e^		3.67 ± 0.27^a^	4.57 ± 0.53^d^	6.72 ± 0.57^d^	5.74 ± 1.24^e^	9.25 ± 0.35^e^
24:0	1.70 ± 0.07^d^	1.08 ± 0.20^c^	1.05 ± 0.02^c^	0.78 ± 0.021^b^	1.07 ± 0.03^c^		1.94 ± 0.02^e^	3.48 ± 0.19^d^	8.07 ± 0.38^d^	10.24 ± 0.42^d^	14.44 ± 1.09^d^		1.14 ± 0.03^c^	3.29 ± 0.43^e^	7.72 ± 0.50^d^	13.14 ± 0.30^d^	13.85 ± 0.48^d^
18:6	2.01 ± 0.06^c^	3.23 ± 0.04^a^	2.16 ± 0.11^a^	1.97 ± 0.17^a^	1.54 ± 0.12^b^		2.91 ± 0.04^c^	17.54 ± 0.43^a^	25.70 ± 0.45^b^	20.53 ± 0.96^c^	26.72 ± 1.28^c^		1.45 ± 0.06^b^	5.43 ± 0.19^d^	11.95 ± 0.77^c^	10.50 ± 0.85^d^	17.43 ± 1.72^c^
12:12	2.30 ± 0.01^b^	2.16 ± 0.15^b^	2.20 ± 0.12^a^	1.95 ± 0.10^a^	1.68 ± 0.05^b^		2.49 ± 0.07^d^	14.93 ± 1.02^b^	24.20 ± 0.88^b^	23.89 ± 0.77^b^	30.77 ± 0.09^b^		1.08 ± 0.04^c^	6.98 ± 0.95^c^	11.00 ± 0.30^c^	12.27 ± 0.53^c^	18.28 ± 0.57^c^
6:18	3.14 ± 0.24^a^	1.27 ± 0.03^c^	1.54 ± 0.02^b^	1.72 ± 0.03^a^	1.87 ± 0.06^a^		1.60 ± 0.04^f^	18.32 ± 0.68^a^	27.74 ± 0.50^a^	32.49 ± 0.93^a^	39.79 ± 1.77^a^		0.51 ± 0.02^d^	14.42 ± 0.53^b^	18.04 ± 0.42^b^	18.93 ± 0.39^b^	21.30 ± 0.95^b^
0:24	1.31 ± 0.01^e^	0.85 ± 0.03^d^	0.83 ± 0.05^d^	1.01 ± 0.03^b^	1.03 ± 0.05^c^		3.63 ± 0.05^b^	14.41 ± 0.72^b^	18.51 ± 1.18^c^	29.03 ± 0.29^b^	31.27 ± 0.36^b^		2.77 ± 0.06^e^	16.97 ± 0.21^a^	22.36 ± 0.81^a^	28.71 ± 0.81^a^	30.54 ± 1.22^a^

The different letters (a, b, c, d, and e) represented significant difference (*p* < 0.05) between the cultures on the same cultivation day

Compared with the commonly used methods of nutritional stress in starch production, adding glucose under circadian rhythm is a competitive method (Table 3). In *Chlorella *sp. AE10, 60.30% dry weight accumulation can be achieved through a two-stage method of large amounts of CO_2_ [31]. However, due to the limitation of nitrogen deficiency on growth, the highest starch productivity could only reach 0.31 g L^−1^ d^−1^. Nevertheless, it is worth noting that adding NaHCO_3_ combined with CO_2_ supply can lead to a starch productivity of 0.5 g L^−1^ d^−1^ in *T. subcordiformis,* mainly due to the high-speed accumulation of dry weight [32]. In *Scenedesmus *sp*.* ASK22, the externally added glucose brought a dry weight accumulation of 4.88 g L^−1^, but starch only accounted for 39.93% of the dry weight, which also limited the yield [33]. In this study, the simultaneous addition of glucose and circadian rhythm can make *P. helgolandica* grow rapidly while accumulating a large amount of starch, and finally obtain a yield of 0.4 g L^−1^ d^−1^. More importantly, the need for less light time helps to save resources under indoor production conditions.

**Table 3:** Comparison of biomass and starch production in microalgae under different culture strategies reported in literatures

	Species	Light–dark cycles	Carbon source	Nutrient stress	Biomass (g L^−1^)	Starch content (% DW)	Starch concentration (g L^−1^)	Starch productivity (g L^−1^ d^−1^)	References
Autotrophy	*Chlamydomonas reinhardtii*	*L*:*D* = 14:10	CO_2_ (5%)	−N	/^a^	12.50	0.06	0.015 (4^b^)	[41]
	*Chromochloris zofingiensis*	Continuous illumination	CO_2_ (1%)	+N (20 mM)	4.29	43.40	1.34	0.27 (5)	[42]
	*Chlorella* sp. AE10	Continuous illumination	CO_2_ (10%)	± N (20 mM/5 mM)	2.80	60.30	1.86	0.31 (6)	[31]
	*Tetraselmis subcordiformis*	Continuous illumination	CO_2_ (2%)	-N + P	1.60	65	1.10	0.37 (3)	[43]
Mixotrophy	*Scenedesmus* sp. ASK22	*L*:*D* = 12:12	Glucose (19.35 g L^−1^)	/	4.88	39.93	1.61	0.23 (7)	[33]
	*Platymonas helgolandica* var. tsingtaoensis	Continuous illumination	Glucose (10 g L^−1^)	/	3.13	29.80	0.93	0.08 (12)	This study
	*Platymonas helgolandica* var. tsingtaoensis	*L*:*D* = 6:18	Glucose (10 g L^−1^)	/	6.35	61.09	3.56	0.40 (9)	This study
Heterotrophy	*Tetraselmis chuii*	Continuous darkness	Glucose (10 g L^−1^)	−N (4.8 mM)	1.00	60.82	0.61	0.02 (30)	[14]
	*Platymonas helgolandica* var. tsingtaoensis	Continuous darkness	Glucose (10 g L^−1^)	+N (10 mM)	2.68	61.82	1.65	0.14 (12)	This study

^a^Data unavailable

^b^The number in the parentheses represented the cultivation day used for calculation and comparison

### The regulation of circadian rhythm causes changes in physiological parameters in *P. helgolandica*

**Fig. 4:**
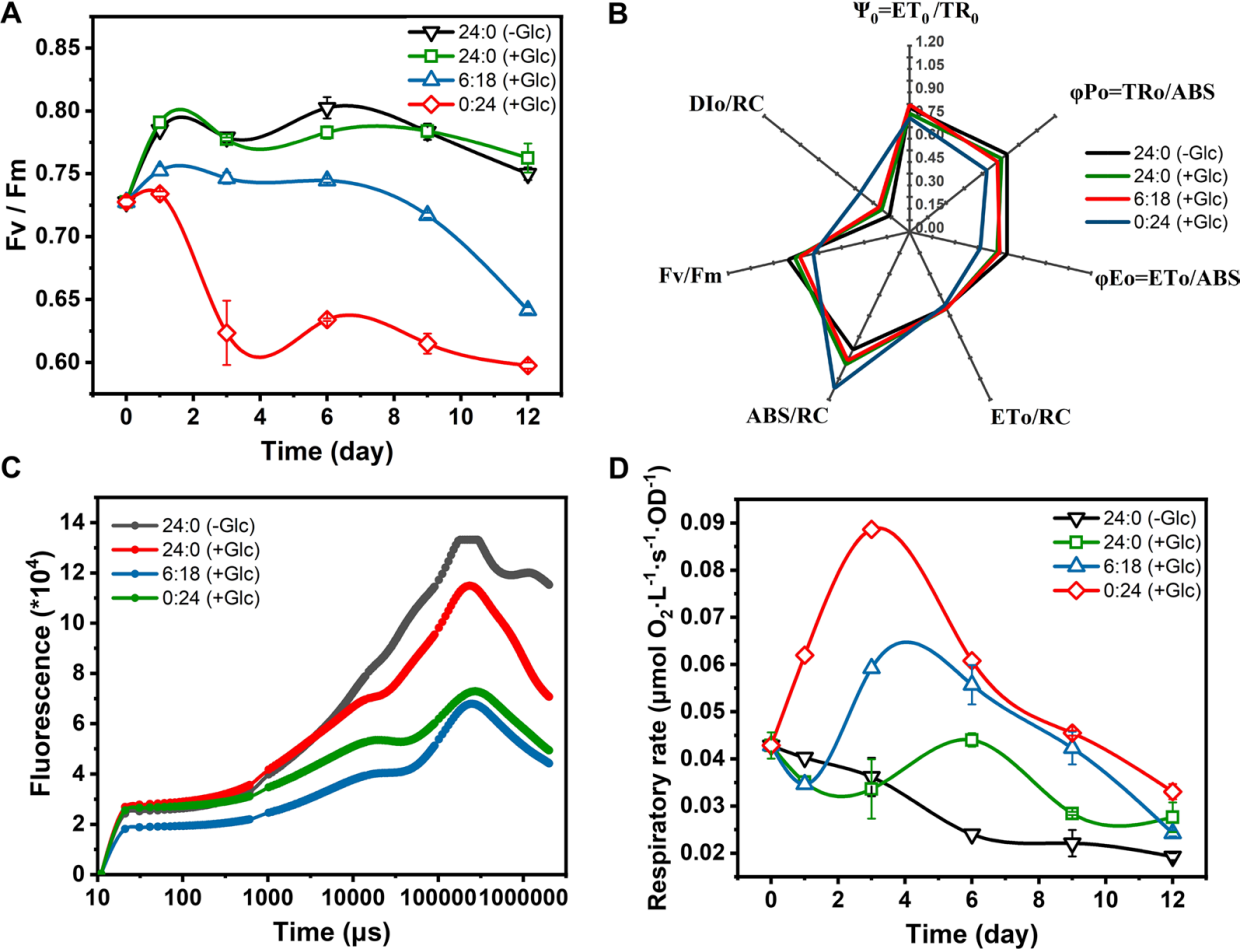
Physiological parameters under different culture modes. Autotrophic group-24:0 (−Glc), mixotrophic group-24:0 (+Glc), circadian group-6:18 (+Glc), heterotrophic group-0:24 (+Glc). **A** *Fv/Fm* changes over time; **B** the spider-plot presentation of selected photosynthetic parameters on 6th day. *Fv/Fm*—optimal/maximal quantum yield of PSII, DIo/RC—heat dissipation per active reaction center, ψ_0_—the efficiency that a trapped exciton can move an electron further than Qa^−^, φ*P*_0_—the maximum quantum yield of primary photochemistry, φ*E*_0_—the maximum yield of electron transport, ETo/RC—the light quantum flux transmitted by the initial electron per active reaction center, ABS/RC—Absorbed light quantum flux per active reaction center; **C** chlorophyll fluorescence kinetic curve; **D** respiratory rate change over time

According to previous experiments, we have determined that with the addition of glucose, applying a day–night cycle of *L*:*D* = 6:18 (h) will enable *P. helgolandica* to achieve the highest starch production rate. We further determined photosynthetic parameters and respiration efficiency of the autotrophic group (24:0, −Glc), the mixotrophic group (24:0, +Glc), the circadian group (6:18, +Glc), and the heterotrophic group (0:24, +Glc), trying to further reveal the differences in the physiological status of *P. helgolandica* under different culture modes. Figure 4A shows the *Fv/Fm* of different groups over time. *Fv/Fm* (PSII maximum electron transfer efficiency) reflects the efficiency of photosystem II (PSII). The overall change trend of mixotrophic group is the same as that of the autotrophic group. The *Fv/Fm* of the circadian group was relatively stable in the early stage of culture, and decreased rapidly in the later stage of culture. Due to the rapidly increasing number of cells, single cell received more limited light. The heterotrophic group consistently maintained very low PSII efficiency.

Figure 4 also shows the multiple parameters of each group measured by the OJIP program on the sixth day (Fig. 4B and C, Additional file 1: Table S2). According to the chlorophyll fluorescence kinetic curve (Fig. 4C), it is found that the curve shape characteristics of the autotrophic group and the mixotrophic group are similar; the curve shapes of the circadian group and the heterotrophic group have changed. Compared with the autotrophic group, their fluorescence level Vj of step J changed by − 10.05% and 33.17%, and the fluorescence level Vi of step I changed by − 25.17% and − 3.40%, respectively. In the end, *Fv*/*Fm* of the two groups were reduced by 9.71% and 20.67%, respectively, reflecting the PSII efficiency decreased with the decrease of the light time.

PI_ABS_ can better reflect the overall performance of photosynthesis. Compared with the autotrophic group, the PI_ABS_ values of the mixotrophic group and the circadian group were lower. It is worth noting that PI_ABS_ of the mixotrophic group was lower than that of the circadian group, which may be due to the increased efficiency of trapped electrons moving to Qa^−^ (ETo/TRo) in the circadian group [34]. The parameters indicating the initial photon absorption flux (ABS/RC) and transmission flux (ERo/RC) of the unit reaction center (RC) in the circadian group did not change significantly compared with the mixotrophic group, and the initial capture light quantum flux per active reaction center (TRo/RC) decreased by 7.10% (*p* < 0.05) compared with the mixotrophic group, which may be caused by the closure of reaction centers in the mixotrophic group due to long-term light exposure. The TRo/ABS and ETo/ABS values of the three groups added with glucose all decreased compared with autotrophic group, indicating that the presence of glucose reduced the capture and transmission of absorbed energy. In the heterotrophic group, the PI_ABS_ value was only 4.353, which decreased by 77.7% and 59.15%, respectively, compared with the autotrophic group and the mixotrophic group. TRo/ABS decreased by 15.98% (*p* < 0.05) compared with the mixotrophic group, DIo/RC increased by 76.52% (*p* < 0.05), indicating that continuous dark culture resulted in a significant decrease in energy capture and a significant increase in energy consumption efficiency. ABS/RC increased by 18.03% (*p* < 0.05) compared with the mixotrophic group, which may reflect that reaction centers in the cells of heterotrophic group were closed in large numbers.

Respiration rates were also tested (Fig. 4D). Compared with autotrophic group, the respiration rate of all the three groups adding glucose increased. The presence of glucose effectively promoted aerobic respiration, released energy, and generated pyruvate for metabolite synthesis. On the 3rd day, the respiration of the heterotrophic group and circadian group were strongly promoted, was 145.12% and 63.67% higher than that of the autotrophic group. This phenomenon shows that in the absence of light, the heterotrophic group maintains growth by up-regulating respiration. In the circadian group, increased respiration rate may indicate more energy production, leading to faster growth.

**Fig. 5:**
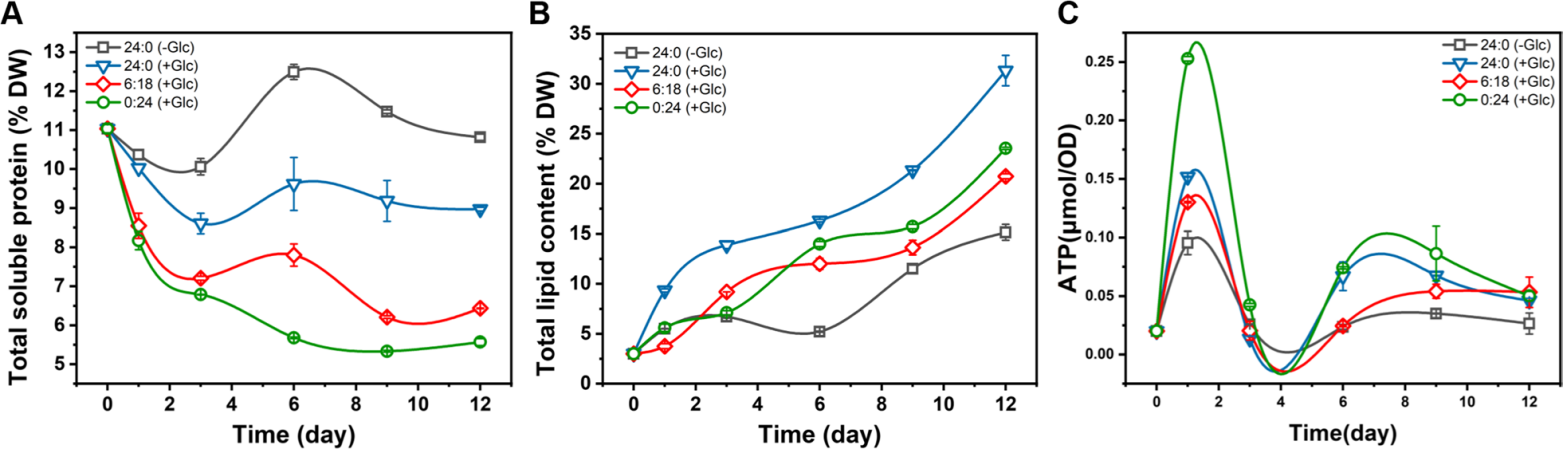
Biochemical components under different culture modes. Autotrophic group-24:0 (−Glc), mixotrophic group-24:0 (+Glc), circadian group-6:18 (+Glc), heterotrophic group-0:24 (+Glc). **A** Total soluble protein content changes over time; **B** total lipid content changes over time; **C** ATP content changes over time

The contents of total soluble protein (TSP), total lipids and intracellular ATP were further determined (Fig. 5). In general, the content of TSP in the autotrophic group was the highest, followed by the mixotrophic group and the circadian group, and the least in the heterotrophic group, which only accounted for about 6% of the dry weight at the end of the culture (Fig. 5A). The total lipid content was accumulated most in the mixotrophic group (the highest proportion of dry weight was 31.32%, on the 12th day), followed by the heterotrophic and circadian groups, and the least in the autotrophic group (Fig. 5B). This indicated that part of energy was used to accumulate lipids instead of starch in the mixotrophic group. The existence of the circadian rhythm led to the reduction of protein and lipid accumulation compared with mixotrophic group. Under culture with circadian rhythm, the biomass components of *P. helgolandica* are close to heterotrophic group, indicating that under long darkness, *P. helgolandica* tends to accumulate starch and reduce protein and lipid synthesis. The intracellular ATP content changed greatly with time (Fig. 5C). The ATP content of the heterotrophic group was maintained at a higher level, which may be due to the fact that the heterotrophic group could generate more ATP by metabolizing glucose through the pentose phosphate pathway in the absence of light. In addition, the cell division of the heterotrophic group was less vigorous than that of the other three groups, so the energy consumption of cell division was reduced (Fig. 2B). At the end of the culture (Day 12), the ATP content of the circadian group was higher than that of the other three groups, while the ATP content of the circadian group was lower than that of the mixotrophic and heterotrophic groups at multiple time points during the culture. A possible explanation is that in addition to synthesizing more starch, the circadian group was also very vigorous in cell division, obtained the largest number of cells during the culture process (Fig. 3A), and cell division is a rather energy-intensive process [23]. By day 12, cell number growth had stopped and energy was mainly used for the accumulation of intracellular products.

### Changes in mRNA levels reflect the influence of culture strategy on the growth and starch synthesis

**Fig. 6:**
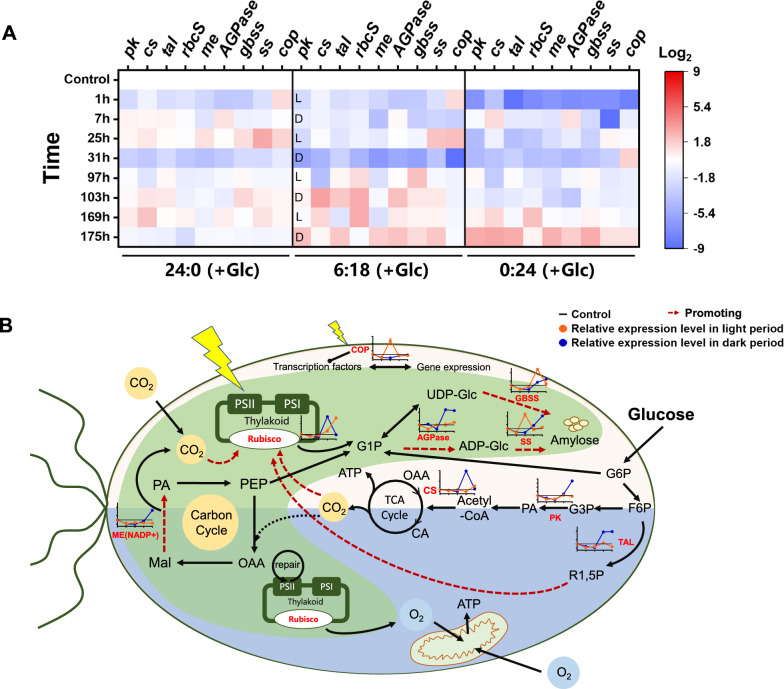
Schematic diagram of starch synthesis under different culture strategies. **A** Relative mRNA levels of 9 genes on Day 0 (1 h,7 h), Day 1 (25 h, 31 h), Day 4 (97 h, 103 h), and Day 7 (169 h, 175 h). Control-24:0 (−Glc). The data represent mean values of triplicates; **B** schematic diagram of starch synthesis. *pk*-pyruvate kinase, *cs*—citrate synthetase, *tal*-transaldolase, *rbcS*-ribulose bisphosphate carboxylase small subunit, *me*-malic enzyme (NADP^+^), *AGPase*-glucose-1-phosphate adenylyltransferase, *gbss*-granule-bound starch synthase, *ss*-starch synthase, *cop*-E3 ubiquitin-protein ligase constitutive photomorphogenesis protein. *L*: light period; *D*: dark period. Line graphs show expression levels at different times

When the cultivation mode is changed, the growth and starch accumulation of *P. helgolandica* have tremendous changes. Both growth and starch synthesis are related to carbon metabolism. In order to explore the metabolic regulation mechanism of algal cells in different modes, the relative mRNA expression levels of 9 genes related to central carbon metabolism pathway, CO_2_ fixation, starch synthesis, and circadian rhythm regulation were determined (Fig. 6A).

After 1 h of culture, the expression of most genes was down-regulated, indicating that the algal cells were influenced when they first entered the glucose-added medium. The time required for the three groups to adapt was different. The mixotrophic group took about 1 day, the circadian group took 4 days, and the heterotrophic group took 7 days, which is basically the same as the trend of cell proliferation and dry weight accumulation (Fig. 3). After experiencing a diurnal cycle, the circadian group upregulated the related gene *cop*. In addition, the circadian cycle group had different gene expression in the light cycle and the dark cycle: the expression of *pk* and *tal* in the dark cycle is increased. The former may indicate increased glycolysis during lack of light, and the latter may represent increased demand for 1,5-diphosphate ribulose synthesis, which is related to cell division and photosynthetic carbon fixation pathways. *rbcS* were continuously upregulated from the 4th day, and correspondingly, the cells began to enter the rapid growth phase at the same time (Fig. 3A). The expression of *me* related to the carbon fixation pathway was relatively high in the dark phase, and it was significantly upregulated on the dark cycle in 7th day. Genes related to starch synthesis continued to be highly expressed after the 4th day. Especially *gbss*, a gene related to amylose synthesis, is in line with the overall trend of starch accumulation (Fig. 3D).

Integrating the determination of mRNA expression levels and physiological parameters (Figs. 4, 6A), we speculated the circadian group promotes growth and starch accumulation through multiple pathways (Fig. 6B). Exogenously added glucose not only promoted central carbon metabolism, leading to more CO_2_, but also promoted the formation of triose (precursors of starch synthesis). The photosynthetic carbon fixation pathway is promoted due to the increase of CO_2_ and the increase of ribulose. The oxygen produced by carbon fixation further promotes aerobic respiration and brought more energy production. In addition, the existence of the dark period contributes to the repair of the photosynthetic system [35], which may have a beneficial effect on the growth of *P. helgolandica*.

## Conclusion

In this study, we proposed a novel regulation method to promote the growth and high amylose starch accumulation by a new isolated Chlorophyta *P. helgolandica*. By adding exogenous glucose and controlling the appropriate circadian light and dark time, the highest dry weight accumulation 6.53 g L^−1^ (*L*:*D* = 12:12 h) can be achieved, and the highest starch concentration could reach 3.88 g L^−1^ (*L*:*D* = 6:18 h). The increased growth rate also helps to reduce the risk of contamination caused by long-term culture with glucose. The highest production rate was 0.40 g L^−1^ d^−1^ after 9 days of production. And this method helps to improve the ability to produce amylose, with the highest accumulation of 39.79% DW amylose. We also discussed the possible mechanism of this phenomenon through revealing changes in the mRNA levels of key genes. The results of this study will help to develop new strategies from microalgae carbon fixation to the production of biologically active substances.

## Methods

### Strain and culture conditions

*Platymonas helgolandica* var. tsingtaoensis HL-1, a marine green microalga, was isolated from the Donghai Sea near Yancheng, Jiangsu Province, PR China, streaking on the plate repeatedly to purify to sterility in the laboratory. Evolutionary tree of 18S rRNA sequence was drawn with MEGA 7. The 18S rRNA sequence of *P. helgolandica* was sequenced by BioMarker, China, sequences of other species were from published sequences in NCBI (https://www.ncbi.nlm.nih.gov/). The microalgae were previously cultivated in KWF medium, with the components as follow (per liter): 30 g sea salt, 0.875 g NaNO_3_, 0.812 g Tris, 0.033 g H_3_BO_3_, 0.0408 g NaH_2_PO_3_, 1.3 mg FeCl_3_, 0.0986 mg (NH_4_)_6_Mo_7_O_24_·4H_2_O, 0.3125 mg CuSO_4_·5H_2_O, 0.4417 mg ZnSO_4_·7H_2_O, 0.3665 mg CoCl_2_·6H_2_O, 0.567 mg MnCl_2_·4H_2_O, 52 mg Na_2_EDTA·2H_2_O, and adjust pH to 7.6 with hydrochloric acid. The culture medium and flasks used in the experiment are sterilized by high-temperature steam (121 ℃, 20 min). The cells were first cultured in a 400-mL glass air bubble column photobioreactor (working volume 300 mL). Sterile air was continuously blown into the medium. The temperature was maintained at 28 ℃, and continuously illuminated from single side with cool white fluorescent lamps that provided an average irradiance of 100 μmol m^−2^ s^−1^. The algae cells were harvested during the late exponential phase and resuspended with fresh KWF ± glucose (2, 5, 10 g L^−1^). The initial cell density was 1.5 × 10^6^ cells mL^−1^ (OD_680_ = 0.3). The culture solutions were divided into 250-mL flasks and placed in a shaker that can start lighting at a fixed time, and the culture was continuously shaken at a speed of 120 rpm. In the experiments under different culture models, the light adjustment ability of the incubator shakers was used to maintain continuous illumination for mixotrophic culture, continuous darkness for heterotrophic culture, and to turn on/off the light at designed time for circadian culture. All experiments were in triplicates.

### Growth measurement

The cell concentration was determined by measuring the absorbance of the algae cultures at 680 nm and counting cells with a hemocytometer and microscope. 5 mL of algae culture was suction filtered onto a piece of dried 0.45-μm filter paper, and dried to a constant weight in a 55 ℃ stove. The weight of the filter paper before and after is measured to calculate the dry weight (DW, g L^−1^). Glucose concentration detection kit (Applygen, China) was used to measure the concentration of glucose in the cultures.

### Biochemical compositions analysis

The cell pellet from 1 to 4 mL of culture was sonicated in 5 mL 95% ethanol on ice. The extracts were centrifuged at 12,000*g* for 1 min, and the absorption of the supernatants at 664 and 648 nm was measured with a spectrophotometer. The chlorophyll content (Chl, mg L^−1^) was calculated with the equation reported in reference [36].

The starch concentration and amylose/amylopectin ratio (Am/Ap) was measured by the methods described by Hovenkamp-hermelink et al. [37]. Simply put, the cells from 0.5 to 4 mL of culture were collected by centrifugation, and washed by 1 mL of 30% perchloric acid to dissolve the starch, which was repeated three times. The supernatant was combined and diluted. The Lugol’s I_2_-KI solution was used to react with the supernatant, and the absorbance at 618 and 550 nm were measured to calculate the concentration of amylose and amylopectin. The concentration of amylose (C_am_, g L^−1^), amylopectin (C_ap_, g L^−1^), and the ratio of amylose and amylopectin (Am/Ap) were calculated according to the standard curve prepared in advance, which was prepared using standard products of amylose and amylopectin. The growth rate (μ, d^−1^), total starch (TS) concentration (X1, g_TS_ L^−1^), amylose concentration (X2, g_Am_ L^−1^), dry weight (X3, DW, g_DW_ L^−1^), starch productivity (Q_X1_, g_TS_·L^−1^ d^−1^), amylose productivity (Q_X2_, g_Am_·L^−1^ d^−1^), total starch content in dry weight (C_TS/DW_, %DW), amylose content in dry weight (C_am/dw_, %DW), amylopectin content in dry weight (C_ap/dw_, %DW) were calculated according to the equations in reference [32, 38].

The total soluble protein was measured by the Bradford method. To describe simply, the soluble protein released by ultrasonicating the cells was dissolved with 0.5 M NaOH, the supernatant was mixed with Bradford reagent, and OD_595_ was measured to reflect the protein concentration. The total lipid was measured by sulpho-phospho-vanillin (SPV) method described by Mishra et al. [39]. The ATP content was measured using ATP content detection kit (Solarbio, China).

Cells from day 9 were collected for transmission electron microscopy (TEM). Cell pretreatment and section preparation were performed according to standard procedures [40]. Cutall microtome LEICA EM UC7 was used to prepare samples. TEM FEI Tecnai Spirit G2 BioTWIN was used to observe.

### Photosystem II (PS II) activity measurement

PS II activity of algal cells was measured by a chlorophyll fluorometer (FluorPen Photon Systems Instruments, Czech). The concentration of algae cultures was adjusted to 20 mg L^−1^ Chl, and performed dark adaption for 20 min. A super pulse at 2100 µmol m^−2^ s^−1^, and a flash at 0.009 µmol m^−2^ s^−1^ were applied to run the OJIP program. Maximal PS II quantum yield was termed as *F*_*v*_*/F*_*m,*_ where *F*_*v*_ represented the variation of chlorophyll fluorescence between maximal fluorescence (*F*_*m*_) induced by saturating pulse and initial fluorescence (*F*_*0*_).

### Respiration rate measurement

Respiration rate was measured by an oxygen meter (FireSting PyroScience, Germany). The concentration of algae cultures was adjusted to OD_680_ ≈ 3, and recovered in light for 30 min. The probe was inserted into the culture medium, and the consumption rate of dissolved oxygen is measured in the dark. The samples were measured every 1 s for 10 min, and the data of the last 5 min were taken to calculate the respiratory rate (μmol O_2_ L^−1^ s^−2^ OD^−1^).

### cDNA synthesis and real time-PCR analysis

RNA was extracted on Day 0, Day 1, Day 4, Day 7, once in light period and once in dark period. Microalgae cultivated in KWF medium with continuous light were used as control. All the RNA samples were extracted by Trizol (Solarbio, China), and cDNA was synthesized using FastKing RT Kit (Tiangen, China). The cDNAs were used for quantitative PCR analysis using M5 HiPer Realtime PCR mix (SYBRgreen) (Mei5bio, China). Target genes were obtained through genome and transcriptome annotation (latest measured by our laboratory, has not been published). The primers were designed with Oligo 7 (Additional file 1: Table S1), and were synthesized by Suzhou Genewiz biotechnology Co., Ltd. The threshold cycle (Ct) values from triplicate reactions were averaged and logarithmically transformed. 18S rRNA was used as internal reference, and all results of treated groups were normalized to the mRNA levels of 18S rRNA.

### Statistic analysis

All the presented data are average values of three biological replications. Error bars indicate the standard deviation. Statistical analysis was performed using SPSS 26.0 for Windows (SPSS Inc., USA), and a value of *p* < 0.05 was regarded as statistically significant.

## Supplementary Information

**Additional file 1: Fig. S1.** Evolutionary tree constructed using 18 s rDNA sequences. Evolutionary tree of 18S rRNA sequence were drawn with MEGA 7. The 18S rRNA sequence of *P. helgolandica* was sequenced by BioMarker, China, sequences of other species were from published sequences in NCBI (https://www.ncbi.nlm.nih.gov/). **Table S1.** Primers in this study for gene expression analysis. **Table S2.** Experimental expressions of the OJIP-test and their calculated values obtained for different cultured group on 6th day (mean ± SD, *n* = 3).

## Data Availability

Data generated or analyzed during this study are included in this published article and its supplementary information files. The genome datasets used during the current study are available from the corresponding author on reasonable request.

## Abbreviations

DW: Dry weight
Am: Amylose
Ap: Amylopectin
Chl: Chlorophyll
TS: Total starch
TSP: Total soluble protein
*F*_*v*_*/F*_*m*_: Maximal PS II quantum yield
TEM: Transmission electron microscopy
PS: Photosynthetic system
Glc: Glucose
*pk*: Pyruvate kinase
*cs*: Citrate synthetase
*tal*: Transaldolase
*rbcS*: Ribulose bisphosphate carboxylase small subunit
*me*: Malic enzyme (NADP^+^)
*AGPase*: Glucose-1-phosphate adenylyltransferase
*gbss*: Granule-bound starch synthase
*ss*: Starch synthase
*cop*: E3 ubiquitin-protein ligase constitutive photomorphogenesis protein
*L*: Light period
*D*: Dark period

## References

[1] Soto-Sierra L, Wilken LR, Dixon CK (2020). Aqueous enzymatic protein and lipid release from the microalgae *Chlamydomonas reinhardtii*. Bioresour Bioprocess.

[2] Li S, Ji L, Shi Q, Wu H, Fan J (2019). Advances in the production of bioactive substances from marine unicellular microalgae *Porphyridium* spp.. Bioresour Technol.

[3] Cai T, Sun H, Qiao J, Zhu L, Zhang F, Zhang J (2021). Cell-free chemoenzymatic starch synthesis from carbon dioxide. Science.

[4] Show KY, Yan Y, Ling M, Ye G, Li T, Lee DJ (2018). Hydrogen production from algal biomass—advances, challenges and prospects. Bioresour Technol.

[5] Liu JZ, Ge YM, Sun JY, Chen P, Addy M, Huo SH (2019). Exogenic glucose as an electron donor for algal hydrogenases to promote hydrogen photoproduction by *Chlorella pyrenoidosa*. Bioresour Technol.

[6] Barati B, Zeng K, Baeyens J, Wang S, Addy M, Gan S-Y (2021). Recent progress in genetically modified microalgae for enhanced carbon dioxide sequestration. Biomass Bioenerg.

[7] Allen J, Unlu S, Demirel Y, Black P, Riekhof W (2018). Integration of biology, ecology and engineering for sustainable algal-based biofuel and bioproduct biorefinery. Bioresour Bioprocess.

[8] Yao C, Ai J, Cao X, Xue S, Zhang W (2012). Enhancing starch production of a marine green microalga *Tetraselmis subcordiformis* through nutrient limitation. Bioresour Technol.

[9] Jiang J, Yao C, Cao X, Liu Y, Xue S (2017). Characterization of starch phosphorylase from the marine green microalga (Chlorophyta) *Tetraselmis subcordiformis* reveals its potential role in starch biosynthesis. J Plant Physiol.

[10] Dammak M, Hadrich B, Miladi R, Barkallah M, Hentati F, Hachicha R (2017). Effects of nutritional conditions on growth and biochemical composition of *Tetraselmis* sp. Lipids Health Dis.

[11] Zachleder V, Brányiková I, Bajpai R, Prokop A, Zappi M (2014). Starch overproduction by means of algae. Algal Biorefineries: Volume 1: cultivation of cells and products.

[12] Morales-Sánchez D, Martinez-Rodriguez OA, Martinez A (2017). Heterotrophic cultivation of microalgae: production of metabolites of commercial interest. J Chem Technol Biot.

[13] Dudek M, Dębowski M, Zieliński M, Nowicka A, Rusanowska P (2018). Water from the Vistula Lagoon as a medium in mixotrophic growth and hydrogen production by *Platymonas subcordiformis*. Int J Hydrogen Energ.

[14] Lu L, Wang J, Yang G, Zhu B, Pan K (2016). Heterotrophic growth and nutrient productivities of *Tetraselmis chuii* using glucose as a carbon source under different C/N ratios. J Appl Phycol.

[15] Bogaert KA, Perez E, Rumin J, Giltay A, Carone M, Coosemans N (2019). Metabolic, physiological, and transcriptomics analysis of batch cultures of the green microalga chlamydomonas grown on different acetate concentrations. Cells.

[16] Lari Z, Abrishamchi P, Ahmadzadeh H, Soltani N (2018). Differential carbon partitioning and fatty acid composition in mixotrophic and autotrophic cultures of a new marine isolate *Tetraselmis* sp. KY114885. J Appl Phycol.

[17] Shen X-F, Qin Q-W, Yan S-K, Huang J-L, Liu K, Zhou S-B (2019). Biodiesel production from *Chlorella vulgaris* under nitrogen starvation in autotrophic, heterotrophic, and mixotrophic cultures. J Appl Phycol.

[18] Azma M, Mohamed MS, Mohamad R, Rahim RA, Ariff AB (2011). Improvement of medium composition for heterotrophic cultivation of green microalgae, *Tetraselmis suecica*, using response surface methodology. Biochem Eng J.

[19] Chin ZW, Arumugam K, Ashari SE, Faizal Wong FW, Tan JS, Ariff AB (2020). Enhancement of biomass and calcium carbonate biomineralization of *Chlorella vulgaris* through Plackett-Burman screening and Box-Behnken optimization approach. Molecules.

[20] Ral JP, Colleoni C, Wattebled F, Dauvillee D, Nempont C, Deschamps P (2006). Circadian clock regulation of starch metabolism establishes GBSSI as a major contributor to amylopectin synthesis in *Chlamydomonas reinhardtii*. Plant Physiol.

[21] Torres-Romero I, Kong F, Legeret B, Beisson F, Peltier G, Li-Beisson Y (2020). *Chlamydomonas* cell cycle mutant c*rcdc5* over-accumulates starch and oil. Biochimie.

[22] Vitova M, Bisova K, Kawano S, Zachleder V (2015). Accumulation of energy reserves in algae: from cell cycles to biotechnological applications. Biotechnol Adv.

[23] Chen T, Liu J, Guo B, Ma X, Sun P, Liu B (2015). Light attenuates lipid accumulation while enhancing cell proliferation and starch synthesis in the glucose-fed oleaginous microalga *Chlorella zofingiensis*. Sci Rep.

[24] Selvakumar P, Umadevi K (2014). Enhanced lipid and fatty acid content under photoheterotrophic condition in the mass cultures of *Tetraselmis gracilis* and *Platymonas convolutae*. Algal Res.

[25] Li T, Yang F, Xu J, Wu H, Mo J, Dai L (2020). Evaluating differences in growth, photosynthetic efficiency, and transcriptome of *Asterarcys* sp. SCS-1881 under autotrophic, mixotrophic, and heterotrophic culturing conditions. Algal Res.

[26] Fang L, Zhang J, Fei Z, Wan M (2020). Astaxanthin accumulation difference between non-motile cells and akinetes of *Haematococcus pluvialis* was affected by pyruvate metabolism. Bioresour Bioprocess..

[27] Seung D (2020). Amylose in starch: towards an understanding of biosynthesis, structure and function. New Phytol.

[28] Li H, Gidley MJ, Dhital S (2019). High-amylose starches to bridge the “Fiber Gap”: development, structure, and nutritional functionality. Compr Rev Food Sci F.

[29] Jobling S (2004). Improving starch for food and industrial applications. Curr Opin Plant Biol.

[30] Dian W, Jiang H, Chen Q, Liu F, Wu P (2003). Cloning and characterization of the granule-bound starch synthase II gene in rice: gene expression is regulated by the nitrogen level, sugar and circadian rhythm. Planta.

[31] Cheng D, Li D, Yuan Y, Zhou L, Li X, Wu T (2017). Improving carbohydrate and starch accumulation in *Chlorella* sp. AE10 by a novel two-stage process with cell dilution. Biotechnol Biofuels.

[32] Qi M, Yao C, Sun B, Cao X, Fei Q, Liang B (2019). Application of an in situ CO2-bicarbonate system under nitrogen depletion to improve photosynthetic biomass and starch production and regulate amylose accumulation in a marine green microalga *Tetraselmis subcordiformis*. Biotechnol Biofuels.

[33] Pandey A, Gupta A, Sunny A, Kumar S, Srivastava S (2020). Multi-objective optimization of media components for improved algae biomass, fatty acid and starch biosynthesis from *Scenedesmus* sp. ASK22 using desirability function approach. Renew Energ.

[34] Aksmann A, Tukaj Z (2008). Intact anthracene inhibits photosynthesis in algal cells: a fluorescence induction study on *Chlamydomonas reinhardtii* cw92 strain. Chemosphere.

[35] Mulo P, Sakurai I, Aro E-M (2012). Strategies for psbA gene expression in cyanobacteria, green algae and higher plants: from transcription to PSII repair. BBA - Bioenergetics.

[36] Hk L (1987). Chlorophylls and carotenoids: pigments of photosynthetic biomembranes. Method Enzymol.

[37] Hovenkamp-hermelink JHM, De Vries JN, Adamse P, Jacobsen E, Witholt B, Feenstra WJ (1988). Rapid estimation of the amylose/amylopectin ratio in small amounts of tuber and leaf tissue of the potato. Potato Res.

[38] Lacroux J, Seira J, Trably E, Bernet N, Steyer J-P, van Lis R (2021). Mixotrophic growth of Chlorella sorokiniana on acetate and butyrate: interplay between substrate, C: N Ratio and pH. Front Microbiol.

[39] Mishra SK, Suh WI, Farooq W, Moon M, Shrivastav A, Park MS (2014). Rapid quantification of microalgal lipids in aqueous medium by a simple colorimetric method. Bioresour Technol.

[40] Ji L, Li S, Chen C, Jin H, Wu H, Fan J (2021). Physiological and transcriptome analysis elucidates the metabolic mechanism of versatile *Porphyridium purpureum* under nitrogen deprivation for exopolysaccharides accumulation. Bioresour Bioprocess.

[41] Gardner RD, Lohman E, Gerlach R, Cooksey KE, Peyton BM (2013). Comparison of CO2 and bicarbonate as inorganic carbon sources for triacylglycerol and starch accumulation in *Chlamydomonas reinhardtii*. Biotechnol Bioeng.

[42] Zhu S, Wang Y, Huang W, Xu J, Wang Z, Xu J (2014). Enhanced accumulation of carbohydrate and starch in *Chlorella zofingiensis* induced by nitrogen starvation. Appl Biochem Biotech.

[43] Yao C, Jiang J, Cao X, Liu Y, Xue S, Zhang Y (2018). Phosphorus enhances photosynthetic storage starch production in a green microalga (Chlorophyta) *Tetraselmis subcordiformis* in nitrogen starvation conditions. J Agr Food Chem.

