# Supplementary material for: Circadian rhythm promotes the biomass and amylose hyperaccumulation by mixotrophic cultivation of marine microalga Platymonas helgolandica
Source: Biotechnol Biofuels Bioprod. 2022 Jul 6;15:75. doi: 10.1186/s13068-022-02174-2 (PMC9261046; doi:10.1186/s13068-022-02174-2)
Supplement: Supplementary file 1 — Additional file 1: Fig. S1. Evolutionary tree constructed using 18 s rDNA sequences. Evolutionary tree of 18S rRNA sequence were drawn with MEGA 7. The 18S rRNA sequence of P. helgolandica was sequenced by BioMarker, China, sequences of other species were from published sequences in NCBI (https://www.ncbi.nlm.nih.gov/). Table S1. Primers in this study for gene expression analysis. Table S2. Experimental expressions of the OJIP-test and their calculated values obtained for different cultured group on 6th day (mean ± SD, n = 3). [file 13068_2022_2174_MOESM1_ESM.docx]

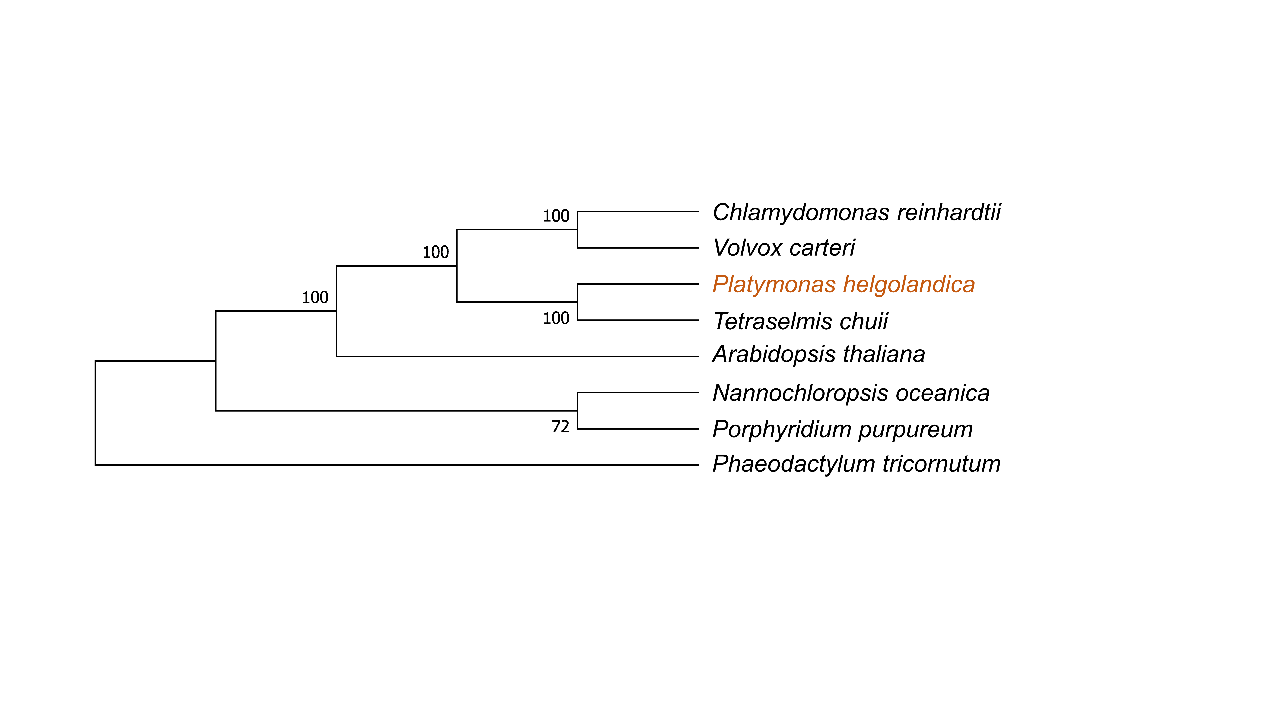


**Fig. S1 Evolutionary tree constructed using 18s rDNA sequences.** Evolutionary tree of 18S rRNA sequence were drawn with MEGA 7. The 18S rRNA sequence of *P. helgolandica* was sequenced by BioMarker, China, sequences of other spices were from published sequences in NCBI (https://www.ncbi.nlm.nih.gov/).

**Table S1 Primers in this study for gene expression analysis**

| Gene | EC number | Primers pairs (5’-3’) |
| --- | --- | --- |
| 18S rRNA | - | F: GGTGTGCACTGGCCAGTCTCATC |
|  |  | R: GAAATACGAATGTCCCCGACTGTCC |
| *pk* | 2.7.1.40 | F: GCTGGACGAGACGCAGGTG |
|  |  | R: GTCGCTTCGGCACGAGTG |
| *cs* | 2.3.3.1 | F: CCGAGCCAGCCCAACCA |
|  |  | R: CGACAGCATGCGCAGCAC |
| *tal* | 2.2.1.2 | F: GAGAAGTGCCGAATTGAGGTGAAG |
|  |  | R: CAGCCCATCCCAAGTCGC |
| *rbcS* | 4.1.1.39 | F: GACCTCCAAGACCACCGACG |
|  |  | R: TGGGCAACGCGCTGGAGATG |
| *me* | 1.1.1.40 | F: CCGAGGATGTGTTTGCTGACGCTG |
|  |  | R: ATTCTTGCGGGGGATGGAGG |
| *AGPase* | 2.7.7.27 | F: CTCTCCGGCGACCACCTC |
|  |  | R: GGTAAGGCATCTCCGCGGC |
| *gbss* | 2.4.1.242 | F: CTTCAAGGAGCTCAACCTGCCA |
|  |  | R: GCCGAATCCAGCTCGACGC |
| *ss* | 2.4.1.21 | F: CCCTCCCCCCCCTCAGG |
|  |  | R: CGAGTACACATGCGTGATTTGGTTCC |
| *cop* | 2.3.2.27 | F: CAGCAGCAGCACATTCAGGCAC |
|  |  | R: CTGTGAGGCTGCACTGGTCGC |

**Table S2 Experimental expressions of the OJIP-test and their calculated values obtained for different cultured group on 6^th^ day** **(mean ± SD, n = 3)**.

|  | 24:0 (-Glc) | 24:0 (+Glc) | 6:18 (+Glc) | 0:24 (+Glc) |
| --- | --- | --- | --- | --- |
| Fo | 23944±1154^b^ | 27715±276^a^ | 18612±71^c^ | 26350±244^ab^ |
| Fj | 43402±3738^ab^ | 48262±764^a^ | 27390±48.5^c^ | 38590±650.5^b^ |
| Fi | 81716±10257^a^ | 74791±1609^a^ | 40151±65^b^ | 52537±780^b^ |
| Fm | 121705±11102^a^ | 114520±2195^a^ | 67541±374^b^ | 72516±179^b^ |
| Fv | 97761±9948^a^ | 86804±1918.5^a^ | 48929±455^b^ | 46166±65^b^ |
| Fv/Fm | 0.803±0.009^a^ | 0.758±0.002^b^ | 0.725±0.003^c^ | 0.637±0.003^d^ |
| Mo | 0.134±0.004^c^ | 0.171±0.001^b^ | 0.120±0.002^c^ | 0.189±0.006^a^ |
| Area | 21637383±1615829^a^ | 23012777±105277^a^ | 13867568±111465^c^ | 17253976±89146^b^ |
| Sm | 225.345±39.459^b^ | 265.267±7.076^b^ | 283.423±0.361^b^ | 373.741±2.457^a^ |
| N | 179.300±27.291^b^ | 190.487±3.720^b^ | 188.941±0.940^b^ | 266.029±0.853^a^ |
| PI_ABS_ | 19.538±0.575^a^ | 10.657±0.061^c^ | 13.071±0.366^b^ | 4.353±0.254^d^ |
| ABS/RC | 0.842±0.013^c^ | 0.948±0.004^b^ | 0.920±0.007^b^ | 1.119±0.000^a^ |
| TRo/RC | 0.676±0.003^b^ | 0.718±0.005^a^ | 0.667±0.003^b^ | 0.712±0.003^a^ |
| ETo/RC | 0.542±0.007^ab^ | 0.549±0.004^a^ | 0.547±0.001^a^ | 0.523±0.008^b^ |
| DIo/RC | 0.167±0.009^d^ | 0.230±0.002^c^ | 0.254±0.005^b^ | 0.406±0.003^a^ |
| ETo/TRo | 0.802±0.007^a^ | 0.764±0.000^b^ | 0.821±0.002^a^ | 0.735±0.009^c^ |
| TRo/ABS | 0.803±0.009^a^ | 0.757±0.002^b^ | 0.724±0.003^c^ | 0.636±0.003^d^ |
| ETo/ABS | 0.644±0.002^a^ | 0.579±0.001^b^ | 0.595±0.003^b^ | 0.468±0.007^c^ |

The different letters (a, b, c, d, and e) represented significant difference (p < 0.05) between the cultures on the same cultivation day

F_0_– the initial fluorescence, Fj– the fluorescence at J-step, Fi– the fluorescence at I-step, Fm– the maximal fluorescence, Fv/Fm-optimal/maximal quantum yield of PSⅡ, Mo– the initial slope of the relative variable fluorescence, Area – the area above the fluorescence curve, Sm – the normalized area above the fluorescence curve, N - QA reduction turnover, PI_ABS_ - Survival index, ABS/RC-Absorbed light quantum flux per active reaction centre, TRo/RC-Initial capture light quantum flux per active reaction centre, ETo/RC-the light quantum flux transmitted by the initial electron per active reaction centre, DIo/RC – heat dissipation per active reaction centre, ETo/TRo-the efficiency that a trapped exciton can move an electron further than Qa^-^, TRo/ABS-the maximum quantum yield of primary photochemistry, ETo/ABS-the maximum yield of electron transport.
